# Development and evaluation of a numerical simulation approach to predict metal artifacts from passive implants in MRI

**DOI:** 10.1007/s10334-021-00966-5

**Published:** 2021-10-16

**Authors:** Tobias Spronk, Oliver Kraff, Jakob Kreutner, Gregor Schaefers, Harald H. Quick

**Affiliations:** 1 Erwin L. Hahn Institute for MR Imaging, University of Duisburg-Essen, Kokereiallee 7, Building C84, 45141 Essen, Germany; 2 High-Field and Hybrid MR Imaging, University Hospital Essen, University Duisburg-Essen, Essen, Germany; 3 MRI-STaR Magnetic Resonance Institute for Safety, Technology and Research GmbH, Gelsenkirchen, Germany; 4 MR:Comp GmbH, Testing Services for MR Safety and Compatibility, Gelsenkirchen, Germany

**Keywords:** Artifacts, Numeric simulations, Metallic implants, Magnetic field strength

## Abstract

**Objective:**

This study presents the development and evaluation of a numerical approach to simulate artifacts of metallic implants in an MR environment that can be applied to improve the testing procedure for MR image artifacts in medical implants according to ASTM F2119.

**Methods:**

The numerical approach is validated by comparing simulations and measurements of two metallic test objects made of titanium and stainless steel at three different field strengths (1.5T, 3T and 7T). The difference in artifact size and shape between the simulated and measured artifacts were evaluated. A trend analysis of the artifact sizes in relation to the field strength was performed.

**Results:**

The numerical simulation approach shows high similarity (between 75% and 84%) of simulated and measured artifact sizes of metallic implants. Simulated and measured artifact sizes in relation to the field strength resulted in a calculation guideline to determine and predict the artifact size at one field strength (e.g., 3T or 7T) based on a measurement that was obtained at another field strength only (e.g. 1.5T).

**Conclusion:**

This work presents a novel tool to improve the MR image artifact testing procedure of passive medical implants. With the help of this tool detailed artifact investigations can be performed, which would otherwise only be possible with substantial measurement effort on different MRI systems and field strengths.

## Introduction

Due to the increasing number of Magnetic Resonance (MR) examinations and the growing percentage of patients with biomedical implants (e.g. stents, orthopedic implants) [1–3], it is becoming increasingly important to obtain a detailed understanding of the behavior of implanted medical devices (IMD) in an MR environment. Apart from the aspects of MR safety, which include testing for displacement forces and torques, as well as radiofrequency (RF) induced heating of medical implants [4, 5], another important aspect in terms of MR compatibility are MR imaging artifacts. MR image artifacts are mainly caused by metallic components of the implants. These components distort the local static magnetic field around the implant and cause incorrect signal localization which leads to signal pileup artifacts. Additionally, signal voids are generated by metallic components due to the rapid phase-coherence loss within one voxel [6, 7]. The overall shape and size of MR image artifacts around medical implants are influenced not only by various parameters like implant-specific parameters (material, geometric shape, size, etc.), but also by MR related parameters (e.g. magnetic field strength, sequence type, echo time and bandwidth). Consequently, a comprehensive investigation requires a large amount of MR measurements to evaluate the influence of each of these parameters on the resulting artifact size [8]. Another consequence is that the size, shape and extent of artifacts caused by metallic implants are hard to predict. In addition this often hampers diagnostic evaluation of potential pathologies in the vicinity of an implant [7]. Various MR sequences have been developed to reduce the artifact size of metal implants (e.g. SEMAC [9], MARS [10], MAVRIC [11]) and to improve MR diagnostics in the vicinity of implants. However, such sequences have the disadvantage of increasing acquisition times. Therefore, a rough estimation of artifact size should be made to decide if such artifact reduction sequences are the right choice of modality and to plan the best sequences for the examination of patients with medical implants.

Due to the clinical relevance of medical implants and the impact of their artifacts on MRI diagnostics, the ASTM F2119 standard has been formulated by the American Society for Testing and Materials (ASTM) defining procedures for measuring MR artifacts from medical implants [12]. This standard describes an experimental setup to determine the artifact size in MRI under strictly defined test parameters. These parameters include the test object and slice orientations, the MR imaging sequences with a clearly defined set of parameters as well as the magnetic field strength [12]. However, the ASTM standard requires performing the testing procedure at only one field strength (1.5T or 3T) and only for a limited set of sequence parameters. This may lead to a limited transferability of test results to all patient cases and potential MRI examinations. If a patient with an implant is to be examined in an MRI system with different field strength, e.g. 3T or even 7T, the results of the artifact test obtained at only 1.5T may have no direct or only limited clinical relevance. Based on the increasing relevance of MR scans at higher field strengths (3T and 7T) [13, 14], currently a potential challenge is that the artifact size of older implants has been evaluated at 1.5T only.

Regarding this context, this study’s aim was to develop and evaluate a numerical approach for simulating the artifact size of several metallic test objects. This numerical approach provides a flexible way of testing artifacts under several different configurations while reducing the actual measuring effort, and hence costs for implant manufacturers seeking MR compatibility certification for their products. MR simulations and measurements of metallic rods made of different materials (test objects) were performed at a field strength of 1.5T, 3T and 7T. Based on the artifact size declared in the implant safety labeling, the numerical approach was also used to develop scaling factors to deduce artifact sizes at different magnetic field strengths. This might be relevant in clinical routines to obtain a rough estimation of the artifact size to be expected under a given magnetic field strength.

## Theoretical background

### Numerical artifact simulation framework

**Fig. 1:**
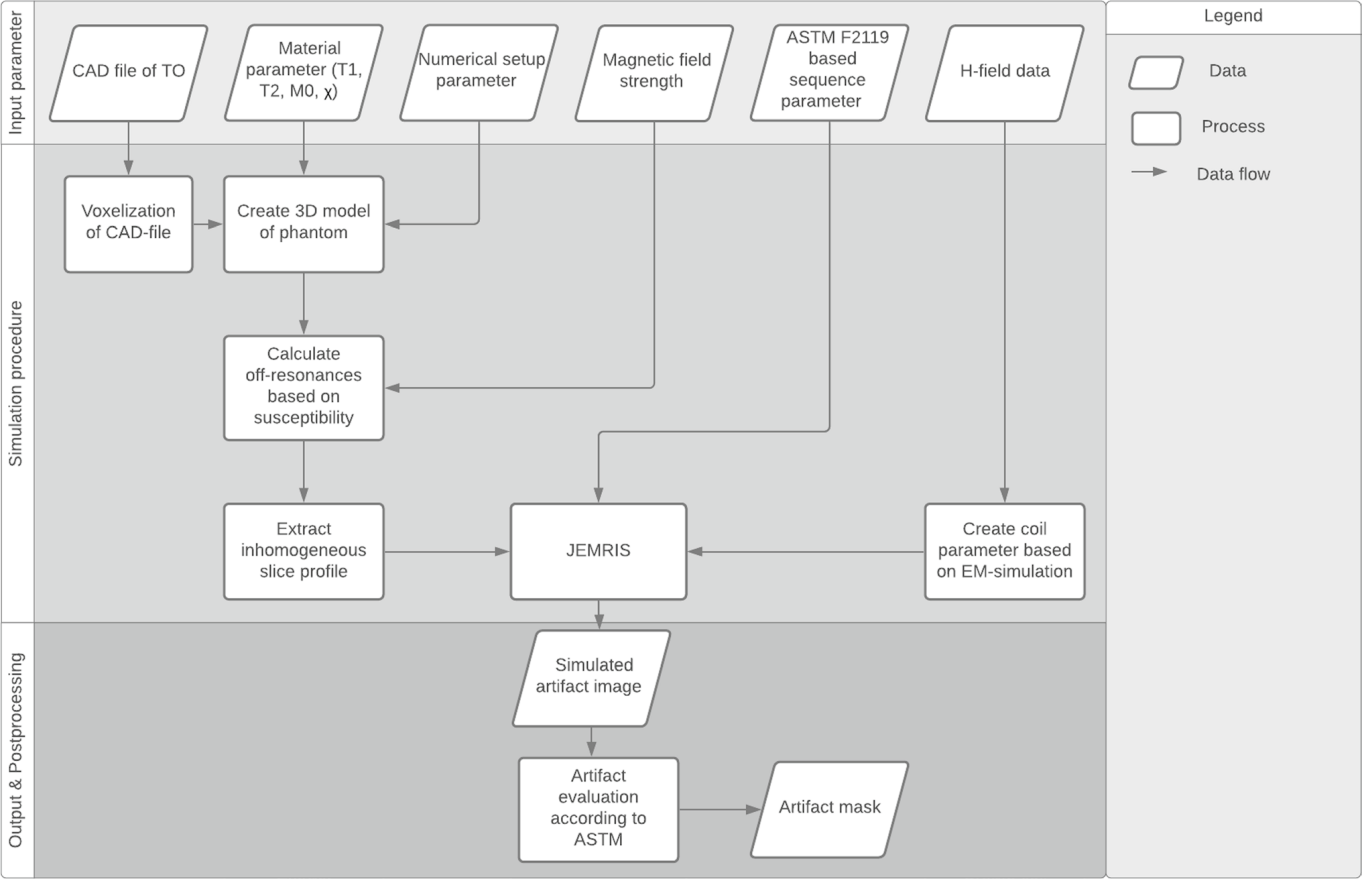
Flow chart of the numerical simulation framework. The framework is split into three general parts which include the definition of the input parameter, the simulation procedure, and the final artifact images with the postprocessing

In general, the artifact size and shape induced by medical implants are influenced by the static magnetic field (*B*_0_), the gradient field and the radiofrequency (RF) field of the MR system. To reduce the complexity of the three different field interactions, the numerical simulation approach in this study is initially limited to the simulation of susceptibility artifacts by the assumption that the artifacts of passive IMDs are essentially dominated by the static magnetic field [6, 15, 16]. These susceptibility artifacts are simulated within a simulation framework that was developed in this study, and that uses the Jülich Extensible MRI Simulator (JEMRIS) [17, 18] as a numerical tool to solve the Bloch equation. Originally, JEMRIS was developed as a tool for sequence development and simulating MRI experiments. In this study, JEMRIS was used for simulating metal-induced MR imaging artifacts. To perform this simulation, our framework was built on top of JEMRIS and includes additional procedures for placing test objects inside the phantom, adjusting MR imaging sequences and evaluating the artifacts according to ASTM F2119 [12]. A general overview of the functionalities of the artifact simulation framework is presented in Fig. 1. All these functionalities are combined in one graphical user interface which allows a fully automatic and flexible numerical artifact investigation for various test objects, sequences, and field strength.

The general structure of the framework is divided into three parts, starting with the definition of the input parameters, followed by the calculation and simulation procedure, and completed by the post processing for artifact evaluation (Fig. 1).

The 3D model of the test object (TO), phantom size and resolution of the numerical model, and material properties for both phantom and test object need to be defined as input parameters. In the first step of the simulation procedure, the 3D surface geometry model of the TO is converted into a volumetric voxel model and placed in the volumetric 3D voxel model of the cubic phantom. This combined model is used to assign material properties, which include the longitudinal relaxation time (T1), transverse relaxation time (T2), proton density (M0) and the susceptibility (*χ*) values of the materials of the TO and the surrounding medium providing background signal. The spatial distribution (*r*) of the susceptibilities (*χ*(*r*)) and the magnetic field strength (*B*_0_) are used to calculate the magnetic off-resonance frequencies *(*$\Delta \delta (r)$*)* caused by the TO [18, 19] (Eq. 1) as follows:

$$\boldsymbol{\Delta }{\varvec{\delta}}({\varvec{r}})={\varvec{\upgamma}}\bullet 2{\varvec{\pi}}\boldsymbol{ }\bullet {{\varvec{B}}}_{0}\boldsymbol{ }\bullet {{\varvec{F}}{\varvec{T}}}^{-1}\left[\left(\frac{1}{3}-\boldsymbol{ }\frac{{{\varvec{k}}}_{{\varvec{z}}}^{2}}{{{\varvec{k}}}_{{\varvec{x}}}^{2}-\boldsymbol{ }{{\varvec{k}}}_{{\varvec{y}}}^{2}-{{\varvec{k}}}_{{\varvec{z}}}^{2}}\right)\boldsymbol{ }\bullet {\varvec{F}}{\varvec{T}}\left({\varvec{\chi}}\left({\varvec{r}}\right)\right)\right]$$

The calculation of the field inhomogeneity is used to simulate through-plane and in-plane artifacts caused by the medical implant. The through-plane artifacts are calculated by considering the inhomogeneous slice profile caused by the medical implant and they are applied to the 3D model to extract a slice with a predefined slice thickness for the subsequent simulation.

The required sequence-specific information for the simulation is created with the JEMRIS sequence development tool which stores the information in a separate file that describes the applied sequence in detail. This allows a flexible adjustment of the sequence parameters such as echo time, repetition time, slice thickness, matrix size, etc. up to the adjustment of gradient strengths or timing of the specific sequence.

In addition to the sequence information, the TO and phantom parameters, the simulation also requires information about the electromagnetic (EM)-field sensitivity of the receiving and transmitting RF coils. For this, H-field data can be included from another EM simulation tool like Sim4Life or ANSYS HFSS. Within the framework of this study, a homogeneous EM field is assumed because the artifacts of the 15-mm sized TOs are mainly dominated by susceptibility differences between TO and background material. This assumption is derived from the results of Song et al. [16] who have shown that small test objects have only minor interactions with the RF field.

With all this information, the original JEMRIS framework solves the Bloch equations and creates the MR image. These simulated MR images are then used to evaluate the artifacts caused by the TO during post processing. Therefore, an artifact mask is created that defines which pixels are declared as an artifact according to ASTM 2119 [12].

## Materials and methods

### Experimental MR imaging setup

**Fig. 2:**
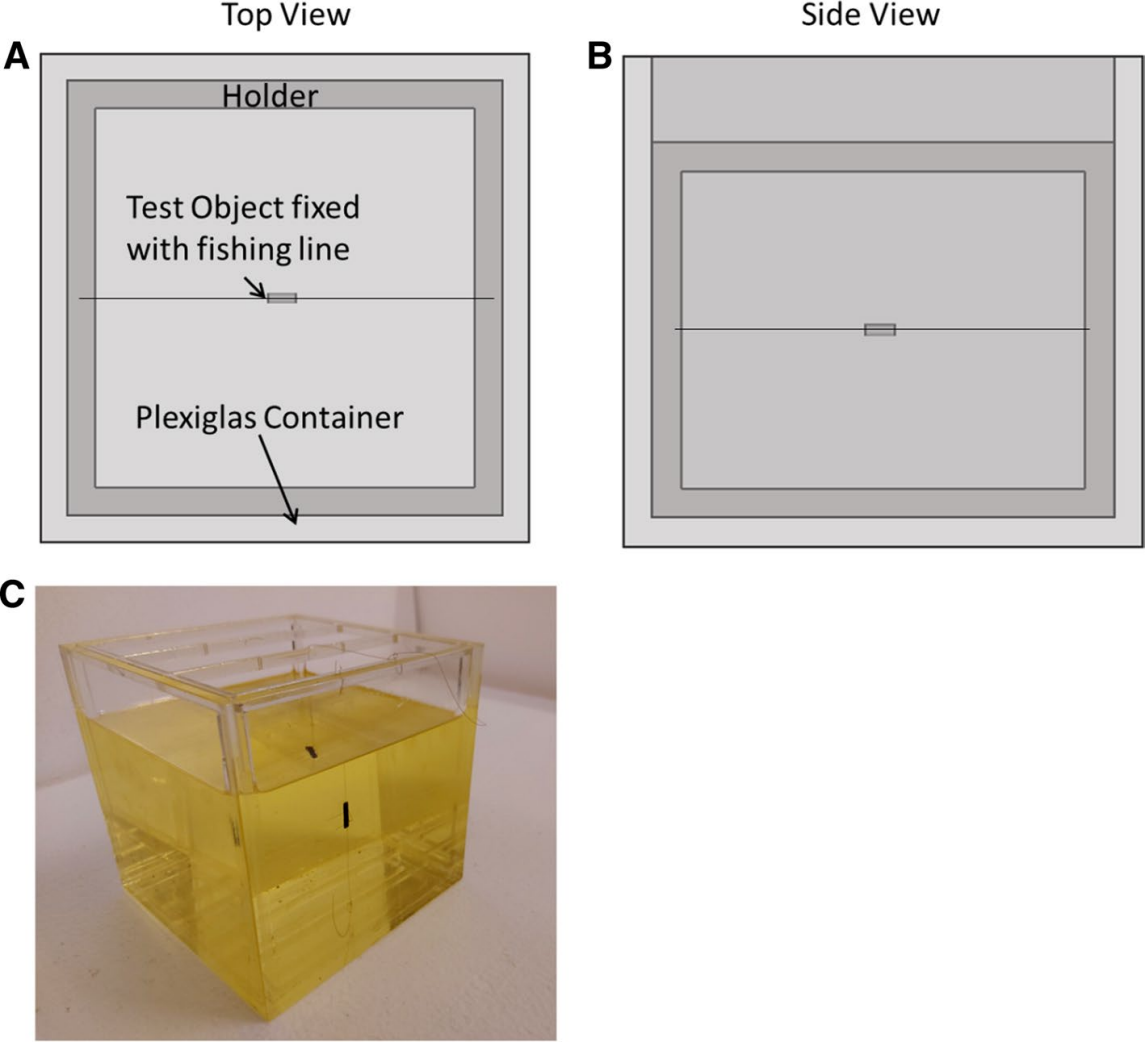
The TOs placed within the holder of the phantom in top view (**A**), side view (**B**), and as a photo (**C**). This holder allows flexible placement of the samples in three orthogonal orientations with regards to the static magnetic field in the MRI systems. The phantom is filled with oil (yellow, transparent) to provide homogeneous background signal

To validate the artifact simulation framework, an experimental MR imaging setup based on the forementioned standard [9] was developed. A cubic Plexiglas container (150 × 150 × 140 mm^3^) filled with 2 L of vegetable oil (*ε* = 0*.*40, $\sigma = 6.6 \frac{\mathrm{mS}}{\mathrm{m}}$) was utilized as a phantom. Oil was used in this study due to its homogeneous signal and high background contrast for all tested field strengths (1.5T, 3T and 7T) and due to its robustness against dielectric effects, even at the 297 MHz excitation frequency at 7T [20]. Overall, four different TOs were investigated in this study, consisting of two metallic and two plastic rods of cylindrical shape each with a diameter of 3 mm and length of 15 mm. The metallic rods were made of titanium (99.5% titanium) and stainless steel (68.9% iron, 18.5% chromium, 8.2% nickel, and 2.2% copper). The 15 mm TO size was selected to reduce the additional effect of gradient and RF-induced artifacts on the overall artifact size. The two plastic rods were used to determine the position of the center slice, as they are clearly visible in MR images and do not generate any artifacts or visual displacement. In order to be able to position the TOs as freely as possible in the phantom, they were individually fixed in the middle of the holder construction by using a fishing line (Fig. 2). Furthermore, this construction allows the TOs to be placed in different orientations relative to the main magnetic field of the respective MRI systems.

The following three MRI systems were used: a 1.5T MAGNETOM Aera, a 3T MAGNETOM Skyra and a MAGNETOM 7T (all Siemens Healthcare GmbH, Erlangen, Germany). The 1.5T and 3T MRI systems were used with a 20-channel RF receiving head coil. The 7T MRI system was used in combination with a 1-channel transmit/32-channel receive head coil (Nova Medical, Wilmington, MA). Due to the lack of an integrated transmit body coil at 7T, the head RF coil for this MR system was equipped with the outer transmit volume birdcage. It thus provides sufficient excitation signal homogeneity of the phantom oil used as background medium, as known from measurements at 1.5 and 3T [20]. Transferability of the experimental setup is given since the Nova Medical RF head coil is widely available at most 7T sites [21] and also proved to provide high reproducibility in multi-center brain imaging studies [22, 23]. The selected MR sequences for all phantom experiments were a gradient echo and spin echo sequence according to ASTM F2119 [12] which are specified in Table 1. For each test configuration, two interleaved image series with an offset of 3 mm were acquired for 24 slices with a slice thickness of 3 mm each. These two-image series were combined in the postprocessing to one gapless image dataset. This was performed to prevent cross talk between slices and to achieve full volume coverage of the TOs in the MR measurements without gaps. The phantom with the different TOs was measured in two different positions relative to the static magnetic field *B*_0_. In the first position, the longitudinal axis of the TOs was oriented parallel to *B*_*0*_. For the second position the longitudinal axis was oriented perpendicular to *B*_0_. For each of the two positions, two different slice orientations were acquired to visualize the shape of the artifact in two directions. One of three possible slice orientations could be omitted because of the rotational symmetric shape of the TOs. Additionally, both possible orientations for the phase-encoding direction were acquired for each slice direction. The whole set of different TO orientations with the different sequences, slice orientations and phase-encoding directions leads to 16 measurements per TO per magnetic field strength.

**Table 1:** This table shows the applied imaging sequences according to ASTM F2119

	Sequences	
	Spin echo	Gradient echo
TE [ms]	20	15
TR [ms]	500	500
Flip angle [°]	90	30
Matrix size	256 × 256	256 × 256
Slice thickness [mm]	3	3
Pixel bandwidth [Hz/px]	130	130
FOV [mm^2^]	200 × 200	200 × 200

In accordance with ASTM F2119, no geometric distortion correction or accelerated imaging acquisitions methods were used

### Numerical setup

The numerical approach for simulating MR image artifacts around medical implants was conceived to achieve the best comparability to the measurement in terms of TO orientation and MR parameters. For the numerical approach, a voxel model of a cubic phantom (150 × 150 × 150 mm^3^) with a spatial resolution of 2 pixels per millimeter was defined. Inside the phantom, a homogeneous background medium was simulated to reproduce the properties of oil from the MR imaging measurement setup (*T*1 = 90 ms, *T*2 = 90 ms, *M*0 = 1, χ = − 8.8 ppm [24, 25]). Within the phantom, each TO was aligned parallel and orthogonal to the direction of the static magnetic field to match the conditions of the experimental setup described above. The material susceptibilities of the TOs are 182 ppm for titanium and 3500 ppm for stainless steel [7].

The applied sequences for the simulations were a spin echo and a gradient echo sequence according to the ASTM F2119 standard and as described for the performed MR measurements. Both sequences acquired the center slice of the TO within the phantom for two orthogonal slice orientations.

### Data analysis

**Fig. 3:**
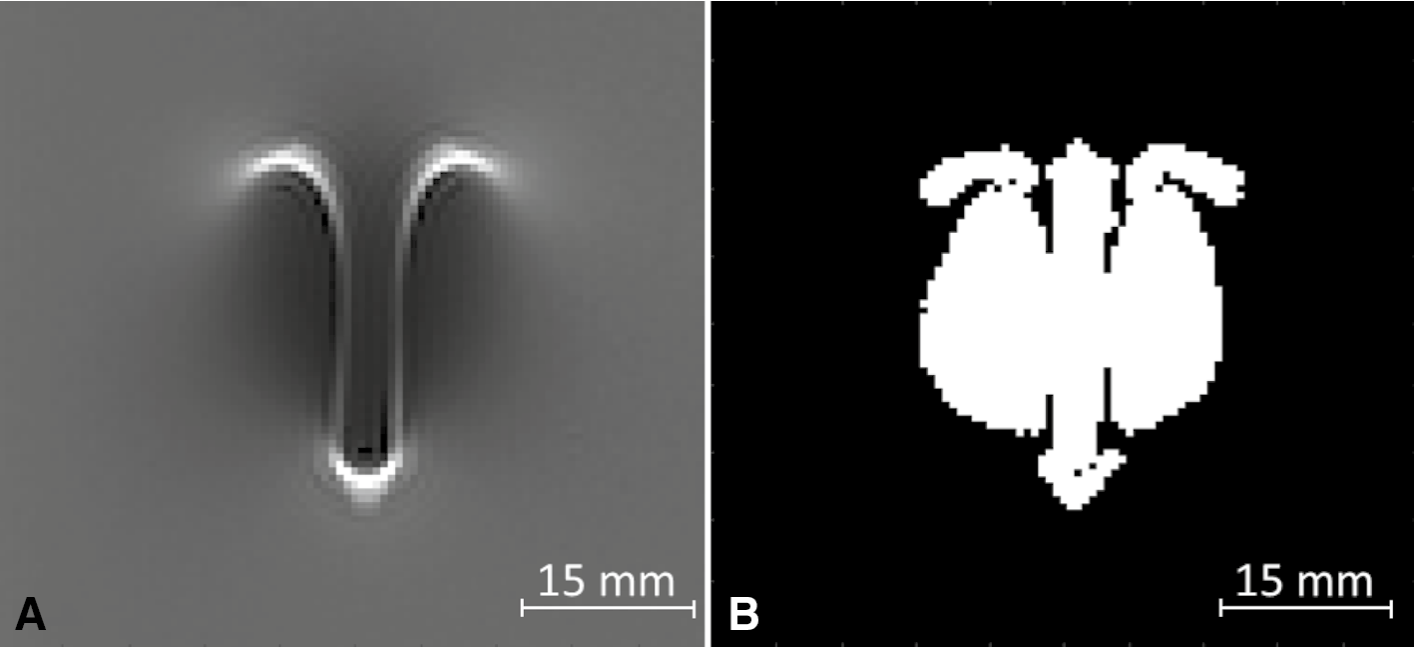
Artifact evaluation: **A** artifact of the titanium rod at 1.5T with coronal slice orientation and vertical TO orientation relative to the static magnetic field; **B** calculated artifact image, which highlights all pixels of the image that show a signal change (positive or negative) of more than 30%

The artifacts of the simulated and measured MR images were calculated for all slices of each TO according to ASTM F2119. According to the standard, an artifact is defined as all pixels with signal change of more than 30% compared to a reference image acquired without the TO in place (Fig. 3) [12]. Signal change between the image with TO and a reference image without TO is determined using the following equation (Eq. 2):

2 $${\varvec{A}}{\varvec{r}}{\varvec{t}}{\varvec{i}}{\varvec{f}}{\varvec{a}}{\varvec{c}}{\varvec{t}}\;\;{\varvec{I}}{\varvec{m}}{\varvec{a}}{\varvec{g}}{\varvec{e}}\left({\varvec{x}},{\varvec{y}}\right)=\frac{{{\varvec{S}}}_{{\varvec{T}}{\varvec{O}}}\left({\varvec{x}},{\varvec{y}}\right)-{{\varvec{S}}}_{{\varvec{r}}{\varvec{e}}{\varvec{f}}}\left({\varvec{x}},{\varvec{y}}\right)}{{{\varvec{S}}}_{{\varvec{r}}{\varvec{e}}{\varvec{f}}}\left({\varvec{x}},{\varvec{y}}\right)},$$

where* S*_*TO*_*(x,y)* defines the signal intensity for each pixel of the image with the TO and *S*_*ref*_*(x,y)* the signal intensity of each pixel of the reference image.

The center slice through the TO was used for artifact evaluation. This slice is the easiest to identify in the measured images and, therefore, allows the most valid comparison of the simulated and measured MR images. The following evaluation process utilizes the total amount of pixels as the artifact size. This creates a data set that allows a separate evaluation of the center slice artifact of different materials under various scanning parameters including field strengths, sequences, slice orientations and phase encoding directions. Within the evaluation, the variation in the artifact size in dependence of other parameters can be shown. A major focus is on the influence of the magnetic field strength on the artifact size. Therefore, the artifact sizes at different magnetic field strengths were evaluated by comparing them with the artifact size of the same test configuration at another field strength.

### Validation of the numerical approach

For the validation of this study, it is necessary to verify the assumptions made for the numerical approach by showing that the simulated artifacts reproduce measurements of real MR image artifacts. To prove these assumptions, it was evaluated whether the size and shape of the artifacts are consistent for the simulated and measured MR images.

**Fig. 4:**
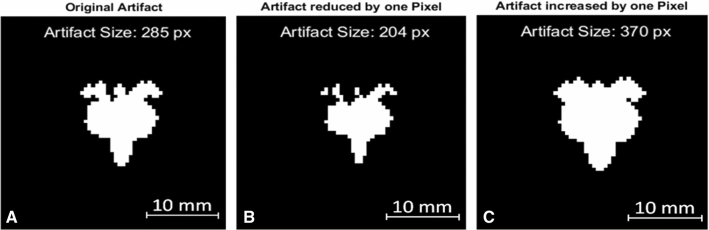
The original artifact from the titanium rod in a spin echo sequence at 1.5T is shown in (**A**). Panel (**B**) shows the eroded artifact (minus one pixel) which creates the minimal artifact size within the uncertainty range. Panel (**C**) shows the dilated artifact (plus one pixel) which creates the maximal artifact size within the uncertainty range

Due to the uncertainty in the MR imaging procedure caused by inhomogeneous magnetic fields and variations in the TO placement, the validation process considered an overall uncertainty of the artifact size. This uncertainty is defined as one pixel (one-pixel uncertainty) in every direction of the artifact which is shown in Fig. 4. Due to the various shapes of the artifacts, it is necessary to perform erosion and dilation to the artifact to estimate the uncertainty of the artifact size. The artifact size of the eroded image (Fig. 4B) will then be used as the negative uncertainty of the artifact size and the dilated image Fig. 4C represents the positive uncertainty of the artifact.

The simulated and measured artifact images of the titanium and stainless steel rods were determined and evaluated according to ASTM F2119. All pixels with a signal change of more than 30% were declared an artifact. In a first validation step the artifact size in the simulation and MR measurement for each test configuration should match. To prove the similarity, the areas of the simulated and measured artifacts were compared and an equivalence test [26] was performed. The equivalence bounds of the test were chosen in correspondence with the averaged one-pixel uncertainty over all simulations and measurements at 1.5T, 3T and 7T. With these, a one-sided *t*-test for the upper and lower equivalence bound and the difference between the simulated and measured artifact size was performed.

In the second step of the validation the artifact shape should match between simulation and measurement as well. In order to verify this, the simulated and measured images of the same test configurations were superimposed based on the center of the artifact. This estimation is necessary because of the large signal losses which prevent an exact determination of the TO position in the measurement. The superimposition allows a pixel-wise evaluation and a determination of the similarity between the measured and simulated artifacts. To quantify the similarity, it is described as the fraction of the congruent pixel divided by all pixels of the artifacts (Eq. 3) as follows:

3 $${\varvec{S}}{\varvec{i}}{\varvec{m}}{\varvec{i}}{\varvec{l}}{\varvec{a}}{\varvec{r}}{\varvec{i}}{\varvec{t}}{\varvec{y}}\boldsymbol{ }\;\;{\varvec{F}}{\varvec{a}}{\varvec{c}}{\varvec{t}}{\varvec{o}}{\varvec{r}}=\frac{{{\varvec{N}}}_{{\varvec{o}}}}{{{\varvec{N}}}_{{\varvec{s}}}},$$

where* N*_*o*_ defines the number of the overlapping pixels between the measured and simulated artifact, whereas *N*_*s*_ describes all pixels that belong to either the simulated or the measured artifact.

### Trend validation

In this section an additional trend analysis is performed in addition to the configuration-based validation of the simulated artifact size and shape. This analysis is used to evaluate configuration-independent trends for the artifact size over the different field strengths. For this purpose, the data was separated by the different field strengths and the artifact sizes were compared to the same test configuration on the other field strengths. This comparison is graphically processed by plotting artifact sizes of the same test configurations in Bland–Altman plots. The error estimation is performed using the one-pixel uncertainty of the artifact sizes. This analysis was conducted separately for the simulated and measured artifact sizes. Overall, the trend analysis compares the artifact size increase between the three different magnetic field strengths, 1.5T, 3T and 7T, and evaluates if the artifact size increase is identical for simulations and measurements.

## Results

### Validation of the numerical approach

**Fig. 5:**
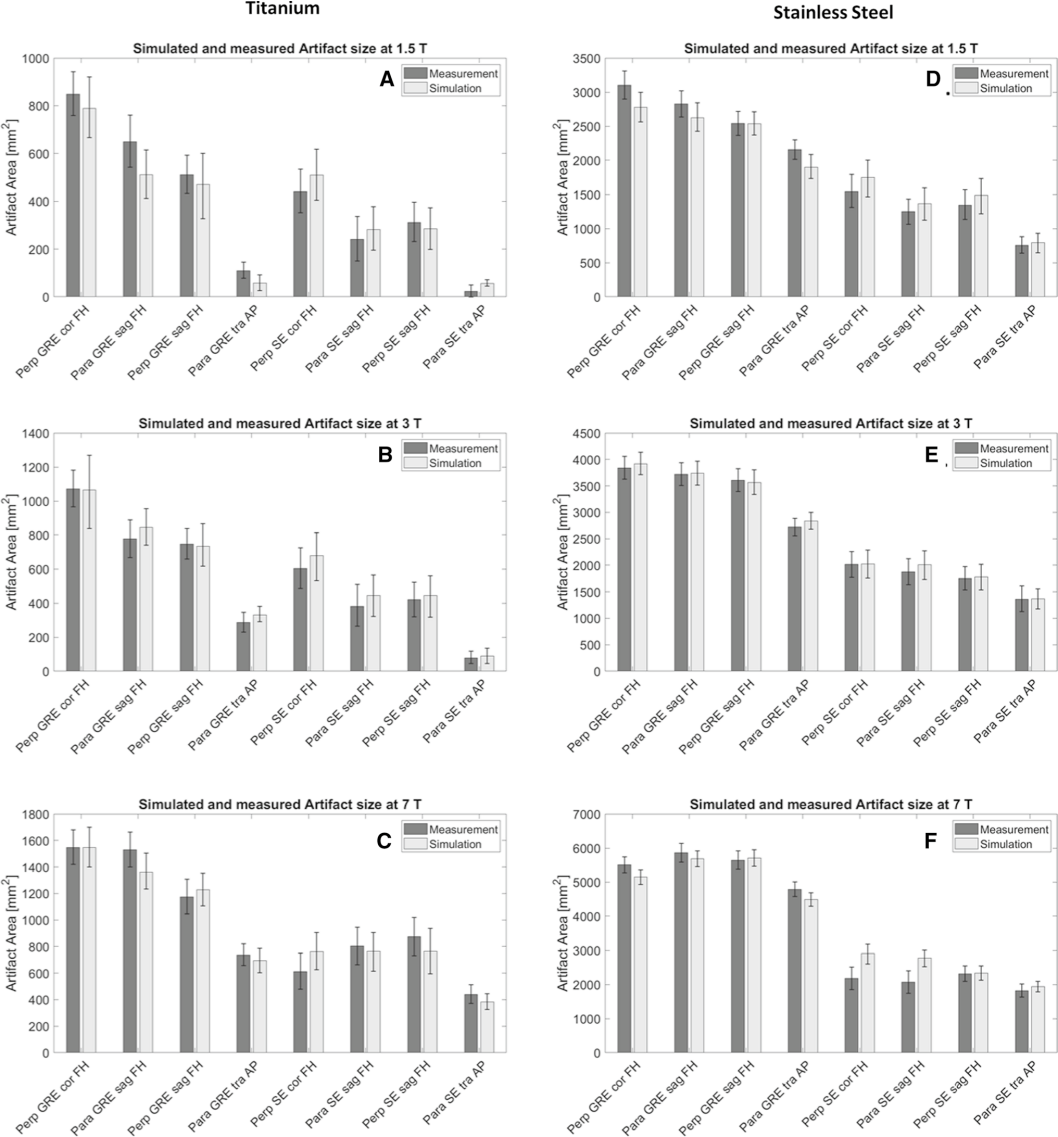
This Figure compares the artifact size of the titanium (**A**–**C**) and the stainless steel rod (**D**–**F**) of the simulations (light grey) and the measurements (dark grey) in mm^2^ separated by the different field strengths, (**A** and **D**) 1.5T, (**B** and **E**) 3T, (**C** and **F**) 7T. Artifact sizes are shown for different implant orientations with regards to *B*_0_ (para and perp), sequences (GRE and SE), slice orientations relative to implant orientation (cor, sag, tra), and phase-encoding direction (FH, AP)

To determine the correlation of the artifact size between simulation and measurement, the corresponding experimental configurations were compared in terms of their artifact size as plotted in Fig. 5. The error bars visualize the one-pixel uncertainty as described in the methods section.

Figure 5 shows that the simulated artifact area corresponds well with the measured artifact area, and the difference between simulated and measured artifact sizes is within the measurement uncertainty. Only the test configuration of the spin echo image at 7T with parallel and perpendicular TO orientation shows larger deviation (Fig. 5C, F). In all graphs it can be observed that the artifact size increases with increasing field strength. Furthermore, all graphs show larger artifacts for gradient echo images compared to spin echo images. The performed equivalence test of the artifact sizes with an equivalence bound of ± 200 pixels between the simulated and measured images also underlines the similarity of the artifact sizes. This test shows that the difference between the simulated and measured artifact sizes lies statistically significant over the lower bound (p_lowerBound_ < 0.005) and under the upper bound (p_upperBound_ < 0.001). Based on these results the equivalence between the two artifact sizes can be estimated with an uncertainty of ± 200 pixels.

The averaged difference between the measured and simulated artifact is  − 35 pixels, meaning that the artifact size of the simulated artifacts is slightly larger than the artifact size of the measured ones.

**Fig. 6:**
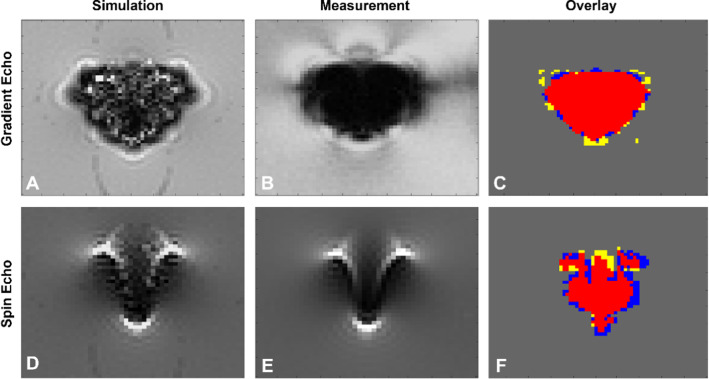
**A** and **D** The simulated spin echo and gradient echo images of a titanium rod acquired on a 3T MRI system. **B** and **E** show the measured spin echo and gradient echo image of the titanium rod. **C** and **F** present overlays of the artifacts from images (**A** and **B**) and (**D** and **E**), respectively. Red color: both artifacts are congruent; yellow: only measured artifact; blue: only simulated artifact

The second validation approach compared the shape of the simulated and measured artifacts. Figure 6 presents these for the spin echo and the gradient echo sequence at 3T. These figures show comparable results for the shape and size of the artifact for the simulated and measured gradient and spin echo images. Furthermore, the similar shape and qualitative distribution of hypointense and hyperintense pixels for the simulated and measured spin echo images can be observed in Fig. 6 as well.

To compare the artifact shape for all configurations, a quantitative evaluation was performed which applied the similarity factor to describe the overlap between measured and simulated images (Fig. 6C, F). The superimposed artifacts of the gradient echo in Fig. 6 show a high similarity factor (0.85) of the simulated and measured images, whereas the spin echo overlay shows a lower similarity factor (0.79) of the two artifacts.

Table 2 presents the averaged similarity factors grouped according to different scanning conditions such as the magnetic field strength and the applied scanning sequence. The similarity factors between simulations and measurements range from 0*.*67 ± 0*.*14 (worst) to 0*.*90 ± 0*.*02 (best) depending on the configuration. The values in Table 2 indicate that the similarity factors between simulations and measurements increase with increasing field strength for both materials. Furthermore, the gradient echo images are characterized by a higher similarity factor compared to the ones generated by the spin echo sequences. The choice of material also affects the similarity between simulation and measurement. The simulations of stainless steel show an 11% higher similarity to the measurement than the simulations of titanium. The averaged similarity factor over all simulations and measurements is 0*.*75 ± 0*.*12 for the titanium rod and 0.84 ± 0*.*12 for the stainless steel one.

**Table 2:** The table shows the averaged similarity factors (with standard deviations) between simulations and MR measurements grouped according to different configurations (field strengths and sequences)

	Similarity factor	
	Titanium	Stainless steel
1.5T	0.67 ± 0.14	0.83 ± 0.07
3T	0.77 ± 0.07	0.85 ± 0.06
7T	0.80 ± 0.09	0.84 ± 0.12
GRE	0.79 ± 0.11	0.90 ± 0.02
SE	0.7 ± 0.11	0.77 ± 0.08
Overall	0.75 ± 0.12	0.84 ± 0.09

### Trend validation

**Fig. 7:**
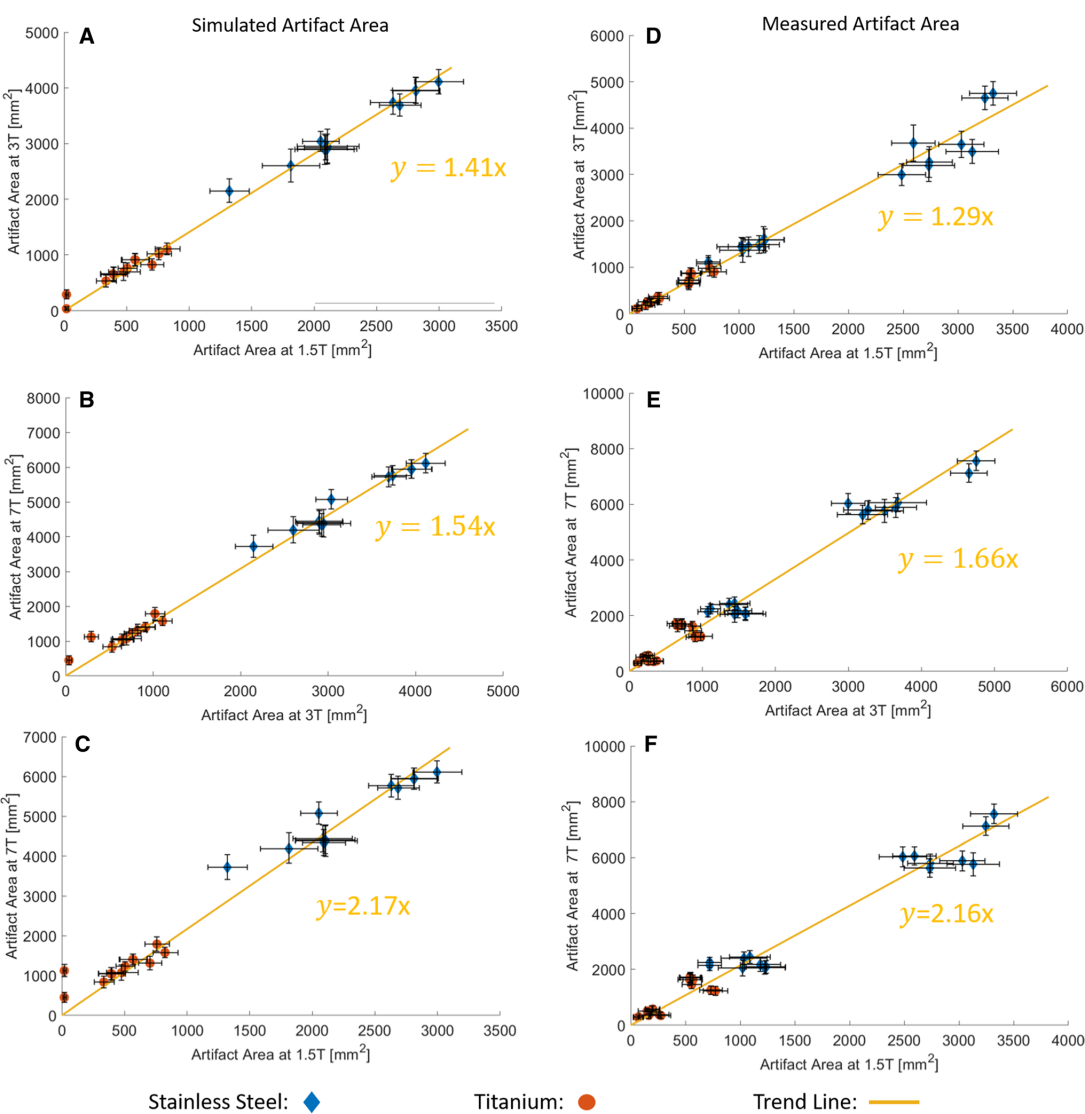
Visualization of the simulated (**A**–**C**) and measured (**D**–**F**) artifact sizes at different field strengths (1.5T, 3T, 7T), each compared to another field strength. Each data point represents a separate test configuration for the different field strengths. Additional trend lines describe the linear correlation and the error bars shows the one-pixel uncertainty for the artifact size. Note that titanium in general provides smaller artifact sizes than implants made from stainless steel

The trend validation provides a more general overview over the artifact sizes of the different materials and field strengths. The comparison of the artifact sizes of the titanium and stainless steel rods at different field strengths demonstrates a strong linear relation between simulated and measured artifact sizes and the magnetic field strength (Fig. 7). This linear correlation is visualized by a trend line, which fits the data with an averaged *R*^2^-value of 0.98 ± 0.01. When comparing the slope of the trend line for the simulated and measured artifact sizes the different configurations do not change more than 9%. Furthermore, the slope of the trend line can be described by the square root of the quotients of the two corresponding field strengths. Based on these results, an equation for a rough estimation of the artifact size between two field strengths can be defined as follows (Eq. 4):

$${\varvec{A}}\left({{\varvec{B}}}_{0,\mathbf{f}\mathbf{i}\mathbf{n}\mathbf{a}\mathbf{l}}\right)=\boldsymbol{ }\sqrt{\frac{{{\varvec{B}}}_{0,\mathbf{f}\mathbf{i}\mathbf{n}\mathbf{a}\mathbf{l}}}{{{\varvec{B}}}_{0,\mathbf{i}\mathbf{n}\mathbf{i}\mathbf{t}\mathbf{i}\mathbf{a}\mathbf{l}}}\boldsymbol{ }}\times \boldsymbol{ }{\varvec{A}}\left({{\varvec{B}}}_{0,\mathbf{i}\mathbf{n}\mathbf{i}\mathbf{t}\mathbf{i}\mathbf{a}\mathbf{l}}\right),$$

where* A* is defined as the artifact area of the different field strengths, *B*_0, initial_ as the field strength with a known artifact area and *B*_0, final_ as the field strength of the target area *A*(*B*_0, final_)*.* Table 3 compares the slopes of the simulated and measured trend lines with the theoretical model. These results show that the simulated slopes (*m*_sim_) and the slopes of the theoretical model (*m*_theo_) differ by less than 0.9%. The measurements also show a maximum difference of less than 11% between the theoretical and the measured slope (*m*_mea_).

**Table 3:** This table compares the slope of the simulated and measured trend lines with a theory-based model

	3T vs. 1.5T	7T vs. 3T	7T vs. 1.5T
Slope *m*_*sim*_ of the simulated trend line (with 95% confidence bounds)	**1.41** (1.38, 1.43)	**1.54** (1.50, 1.58)	**2.17** (2.09, 2.25)
Slope *m*_*mea*_ of the measured trend line (with 95% confidence bounds)	**1.29** (1.24, 1.33)	**1.66** (1.59, 1.73)	**2.16** (2.04, 2.24)
$\frac{{B}_{0, higher}}{{B}_{0, lower}}$	2.00	2.33	4.67
${m}_{theo}=\sqrt{\frac{{B}_{0, higher}}{{B}_{0, lower}}}$	1.41	1.53	2.16
Difference between m_sim_ and $\sqrt{\frac{{B}_{0, higher}}{{B}_{0, lower}}}$	0%	0.9%	0%
Difference between m_mea_ and $\sqrt{\frac{{B}_{0, higher}}{{B}_{0, lower}}}$	− 10.8%	6.7%	− 4.6%

The first two rows of the table show the simulated and measured slope (bold) from Fig. 7 with the positive and negative limits (values in the brackets) at a confidence interval of 95%

## Discussion

The purpose of this study was to develop and evaluate a numerical approach for simulating the artifact size of several metallic TOs for various configurations and across magnetic field strengths ranging from 1.5T to 7T. Starting with the original JEMRIS numerical tool, our framework was extended by procedures to setup the implants inside the phantom, adjusting MR imaging sequences and evaluating artifacts according to ASTM F2119. In addition, effects like an inhomogeneous slice profile caused by high susceptibility differences between the background medium and TOs were also included in our framework. In the first part of the investigation, simulations and MR measurements were compared to demonstrate that the simulation tool delivers realistic results. For this purpose, simulated images were overlaid with measured MR images. Both the size and shape of the simulated artifacts were congruent to the measured ones which was demonstrated by the pixel-wise comparison. This comparison of simulation and measurement showed that the artifacts match in shape as well as in size with an overall similarity factor of 0.80 over all testing configurations and field strengths. Based on this high similarity it can be stated that the general assumptions made for the numerical approach were valid for the chosen TOs that had a simple shape and small volume. Small TOs are mainly dominated by susceptibility artifacts, while the influence of gradient and RF induced artifacts can be neglected [16, 27]. However, the simulated gradient echo images showed some bright areas and structures within the otherwise dark artifact that we did not observe in the MR measurements. This phenomenon in the simulated images can be explained by a numerical error which is caused by the rapid intra-voxel dephasing in this area. However, this phenomenon did not have a quantitative effect on the subsequent evaluation because these bright areas were also recognized as an artifact.

In the second part of the investigation, the artifact areas of different materials were compared over different field strengths for the simulations and measurements. This comparison demonstrated that the change of the artifact area between field strengths follows a linear correlation showing increasing artifact sizes with increasing field strengths. The slope of this linear correlation only depends on two field strengths, thus allowing interpolation or extrapolation of the results to further field strengths. Previous studies also showed a strong correlation between the MR image artifacts from metallic objects and the magnetic field strength, but they did not quantify the correlation, nor deduced a scaling factor [6, 8]. One other study quantified the change of the artifact size over different field strengths by defining the artifact size as the distance from the edge of the implant to the fringe of the artifact [28]. This characterization of an artifact mainly includes the signal shift in the frequency-encoding direction. In contrast, the evaluation of the artifact area, which was performed in our study, also includes the effects of intravoxel dephasing [29]. This may explain the different behavior of the artifacts between the different field strengths in the two studies. However, the description of artifact growth over a single dimension is unintuitive because it is difficult to apply to an artifact image. An artifact area is better suited to achieve this because it can be scaled easily and a better overview over the whole artifact can be deduced. The obtained relation between the artifact size and the magnetic field strength can be helpful for clinical staff for a rough estimation which artifact size can be expected at other field strengths.

It must be noted that the choice of RF coils in this study did not have any direct impact on the simulated and measured artifact sizes. For practical reasons, RF head coils were chosen for all three MRI systems involved. All three RF head coils were similar in design for signal reception. The phantom was designed to fit into all three RF head coils and to allow for different orientations of the phantom regarding the main magnetic field during the measurements. Since simulated and measured artifact sizes proved to be independent of the choice of RF coil, the results from this study are also valid for small passive implants that may be located outside the head/neck region elsewhere in the human body (e.g. surgical and vascular clips, surgical screws and fixation plates).

A limitation of this study is that the numerical approach was only proven for susceptibility dominated TOs which allows an application only to smaller passive implants mentioned above. This may lead to systematic underestimation of the artifact size of larger implants additionally influenced by the RF transmit and gradient fields [27]. One additional challenge of the numerical approach is the determination of the exact susceptibility of materials to be simulated. The susceptibility of stainless steel can fluctuate strongly between different TOs because of the varying components of the steel alloy (iron, chromium, nickel) and their volume fractions within the alloy. Without an exact determination of the susceptibility, a realistic simulation is not possible. However, the compositions should be known to the manufacturer of the implants. In addition, an iterative MR-based method [30] can be used to determine the susceptibility of the TOs before the numerical simulation. Another limiting factor is that a completely homogeneous static magnetic field cannot be achieved by the MR system during measurements. This may explain the increased difference in the artifact simulation for the spin echo sequences at 7T. But even with the increasingly inhomogeneous *B*_0_ field at higher field strengths, the artifacts can be simulated for the 7T spin echo with a similarity factor of 0.7 for titanium and 0.77 for stainless steel. To further maximize the similarity factor in upcoming simulations, the *B*_0_ field distribution of the MR system could be measured and then used in the numerical approach.

In future applications the numerical approach for MR artifact simulation will also be applied to more complex and larger passive medical implants such as implants for osteosynthesis and joints that otherwise require a larger MR measuring effort.

## Conclusion

This work presents a novel tool to improve the MR image artifact testing procedure of passive medical implants. With the help of this tool detailed artifact investigations can be performed, which would otherwise only be possible with substantial measurement effort on different MRI systems and field strengths. The evaluation of the artifact size over different magnetic field strengths shows that it is straightforward to determine and predict the artifact size for other field strengths based on a single MR measurement at one field strength.
